# Cytokine concentration and T cell subsets in the female genital tract in the presence of bacterial vaginosis and *Trichomonas vaginalis*

**DOI:** 10.3389/fcimb.2025.1539086

**Published:** 2025-04-17

**Authors:** Marisa R. Young, Lisa B. Haddad, Lyle McKinnon, Walter O. Ochieng, Marta Rowh, Amanda Gill, Igho Ofotokun, Supriya D. Mehta

**Affiliations:** ^1^ Department of Gynecology and Obstetrics, Emory University School of Medicine, Atlanta, GA, United States; ^2^ Center for Biomedical Research, Population Council, New York, NY, United States; ^3^ Department of Medical Microbiology and Infectious Diseases, University of Manitoba Max Rady College of Medicine, Winnipeg, MB, Canada; ^4^ Office of the Director, Center for Global Health, United States Centers for Disease Control and Prevention, Atlanta, GA, United States; ^5^ Department of Emergency Medicine, Emory University School of Medicine, Atlanta, GA, United States; ^6^ Emory University School of Medicine, Atlanta, GA, United States; ^7^ Divison of Infectious Diseases, Department of Medicine, Emory University School of Medicine, Atlanta, GA, United States; ^8^ Division of Infectious Diseases, Department of Medicine, Rush University College of Medicine, Chicago, IL, United States; ^9^ Division of Epidemiology and Biostatistics, School of Public Health, University of Illinois at Chicago, Chicago, IL, United States

**Keywords:** *Trichomonas vaginalis*, bacterial vaginosis, inflammation, cytokines, T cells, HIV risk, vaginal mucosa, female genital tract

## Abstract

*Trichomonas vaginalis* (TV) and bacterial vaginosis (BV) are highly prevalent vaginal infections. Both are associated with pelvic inflammatory disease and HIV acquisition and transmission, though the underlying mechanisms are incompletely understood. We characterized the effect of TV and BV infection on inflammatory markers in the vagina among reproductive-aged women in Atlanta, Georgia. Cervicovaginal lavage specimens were collected from HIV seronegative women at a baseline visit and again three months later. Eighteen individual cytokines, 17 T cell subsets, BV, and TV were measured at both timepoints. After natural log transformation, the median cytokine concentration and number of T cells were compared by infection status statistically using the Kruskal-Wallis test. A cytokine inflammation score and a T cell score were created using principal components analysis. The scores were then used as outcomes in separate linear mixed regression models with a random intercept. Sixty women had baseline data and 43 were seen for follow-up. The median age was 30 years, 78% self-reported Black race. TV and BV prevalence at the baseline visit was 15% and 37%, respectively. The concentration of 16 out of 18 cytokines differed by infection status. In multivariable modeling, neither TV nor BV were associated with cytokine score. Most CD4+ T cell subsets (7 out of 9) differed by infection status. In a multivariable model, TV infection was associated with a higher T cell score (1.54; 95%CI 0.00, 3.08). BV was not associated with a higher T cell score. Increased concentration of vaginal mucosal T cells may explain the observed association between TV infection and HIV risk.

## Introduction

Vaginal complaints including malodor and abnormal discharge are among the most common reasons for seeking gynecologic care (Anderson et al., 2004). Bacterial vaginosis (BV) and *Trichomonas vaginalis* (TV) are two of the most prevalent etiologies of vaginitis. The estimated prevalence of TV in US women aged 15-59 is 2.1% (Flagg et al., 2019) with 3.5 million incident cases in this age group in 2018, the most recent year for which these data are available (Lewis et al., 2021). Prevalence estimates for TV are less certain, since trichomoniasis is not a reportable infection (Lewis et al., 2021). General population prevalence of BV has been estimated to range between 23-29% globally (Peebles et al., 2019), however prevalence is highly variable by geographic region, race and ethnicity. Beyond the management of symptoms and associated healthcare costs, TV and BV are associated with a variety of adverse reproductive health outcomes even among asymptomatic individuals including: pelvic inflammatory disease and infertility (Wiringa et al., 2019; Ravel et al., 2021), preterm birth (Van Gerwen et al., 2021; Gudnadottir et al., 2022), and increased risk of HIV acquisition and transmission (Kissinger and Adamski, 2013; Mtshali et al., 2021a). BV and TV are frequently co-occurring in the vagina, where 40-60% of TV positive individuals are also BV positive (Simhan et al., 2007; Gatski et al., 2011; Huang et al., 2023). The mechanistic link between infection and the notable increased risk of adverse clinical outcomes, however, is incompletely understood. Understanding how vaginal infections influence specific cytokine and cellular immune responses may help elucidate the mechanism by which they contribute to adverse health outcomes.

The immune system and inflammation likely contribute to the effect of vaginal infections on the adverse reproductive health outcomes detailed above. Several prior studies have shown increased cytokine concentrations, especially IL-8, but also MIP3α, and sTNFr1 with TV infections *in vitro* (Fichorova et al., 2006; Fichorova et al., 2013) and *in vivo* (Simhan et al., 2007; Jarrett et al., 2015). BV has been associated with multiple elevated cytokine concentrations, but especially IL-1β (Dabee et al., 2021; Mtshali et al., 2021b). BV has been associated with increased expression of CCR5+ T cells in the genital tract in some studies (Thurman et al., 2015; Dabee et al., 2019), however this has not been consistently demonstrated in all studies (Mitchell and Marrazzo, 2014; Shannon et al., 2017). There are few studies describing the distinct cytokine and T cell profile associated with specific vaginal infectious etiologies and several studies analyze immune profile in relation to sexually transmitted infections (STIs) as a composite of multiple etiologies (Mlisana et al., 2012; Jarrett et al., 2015). The objective of this study was to characterize the independent effect of TV and BV infection on inflammatory makers in the vagina among reproductive-aged women enrolled in a cohort study in Atlanta, Georgia.

## Method

Data for this analysis come from a prospective cohort examining the impact of progestin contraceptive initiation (Etonogestrel implant, Levonorgestrel intrauterine device, depot-medroxyprogesterone acetate injection) on HIV target cells and inflammatory markers in the lower genital tract (Haddad et al., 2020). Cis-gender women interested in initiating one of the contraceptive methods were recruited from Grady Memorial Hospital clinics or fliers posted in the Atlanta area. Eligibility criteria included: age 18-45 years, normal length of three prior menstrual cycles (defined as 22 to 35 days); HIV seronegative (rapid testing via OraQuick™); not using a copper intrauterine device or hormonal contraceptive for six months prior to enrollment; medically eligible to initiate desired contraception (according to the US Centers for Disease Control and Prevention Medical Eligibility Criteria (Curtis et al., 2016)); and no signs or symptoms of cervicitis on examination (as determined by examining clinician) at the time of enrollment.

Each participant was scheduled for four study visits. The first visit was scheduled to coincide with the luteal phase of the menstrual cycle and the second visit during the follicular phase. The contraceptive method of choice was initiated at the completion of the second visit. The third visit was scheduled approximately three months after visit two, and the fourth visit was scheduled two weeks later. Pelvic exam and specimen collection (as described below) were conducted at all four visits. If the participant reported bleeding on the day of their appointment, they were rescheduled for after bleeding resolved.

At the baseline visit, a questionnaire was administered, and data were collected on participant’s demographic characteristics, prior contraception history, medication use, intravaginal practices (such as douching or application of products to the vagina), substance use, reproductive/sexual/menstrual history, and medical and STI history. At follow-up visits, a short questionnaire related to recent medication exposures and sexual history was administered. Ethical approval for this study was obtained from the Emory University Institutional Review Board and the Grady Memorial Hospital Research and Oversight Committee.

### Specimen collection

During study visits, pelvic examination was performed to collect cervicovaginal swab and cervicovaginal lavage (CVL) samples from participants. After insertion of a speculum without gel, a cervicovaginal swab specimen was obtained for STI testing (DrySwab™, Lakewood Biochemical Company). Following this, CVL samples were collected. Ten milliliters of phosphate-buffered saline was instilled into the vagina and a pipette used to continuously irrigate the posterior fornix, vaginal walls and cervix for 60 seconds using a standardized protocol described by the Microbicide Trials Network (Swaims-Kohlmeier et al., 2016). A second CVL specimen was then collected in an identical fashion. CVL supernatant was obtained after performing Percoll gradient centrifugation on samples. Blood was collected in 8mL sodium citrate containing CPT™ tubes (BD Biosciences) and transported to the laboratory on ice within four hours of collection. Tubes were centrifuged to separate plasma from peripheral blood mononuclear cells (PBMCs) according to the manufacturer’s instructions. Plasma and CVL supernatant aliquots were stored at -80°C until analysis.

### Cytokines

The following pro-inflammatory, anti-inflammatory, and chemotactic cytokines were tested in CVL supernatant (Luminex technology with xPONENT software™, Luminex Corporation): Granulocyte colony-stimulating factor (GCSF), Granulocyte-macrophage colony-stimulating factor (GMCSF), Fractalkin, interferon alpha-2 (INFa2), Interferon gamma (INFγ), Interleukin 12p70 (IL-12p70), soluble CD40 ligand (sCD40L), IL-17a, IL-1a, IL-1b, IL-2, IL-4, IL-6, IL-8, interferon-gamma inducible protein 10 (IP10), macrophage inflammatory protein 1a (MIP1a), MIP1b, and Tumor necrosis factor alpha (TNFα).

### T cells

The following cellular immune markers were tested in CVL by flow cytometry: CD4+, CD8+ (cytotoxic T cells), tissue resident memory like cells (TRM-like; CD4+CD103+ and CD8+CD103+), CD4+ T cells expressing CCR5, the HIV co-receptor (CD4+CCR5+), central memory T cells (TCM; CD4+CD45RA^lo^CCR7^hi^ and CD8+CD45RA^lo^CCR7^hi^), effector memory T cells (TEM; CD4+CD45RA^lo^CCR7^lo^ and CD8+CD45RA^lo^CCR7^lo^), terminally differentiated effector memory cells (TEMRA; CD4+CD45RA^hi^CCR7^lo^ and CD8+CD45RA^hi^CCR7^lo^), and activated T cell subsets (CD4+CD38+, CD4+CD38+HLA-DR+CCR5+, CD4+CD38+HLA-DR+CCR5-, CD8+CD38+, CD8+CD38+HLA-DR+CCR5+, CD8+CD38+HLA-DR+CCR5-).

### Primary exposure of interest

Infection with TV, *Neisseria gonorrhoeae* (NG), and *Chlamydia trachomatis* (CT) was determined using Qiagen Rotor-Gene Q real-time PCR. Bacterial vaginosis was determined by Nugent’s criteria using gram stains prepared from CVL. BV was considered present with Nugent score ≥7. Due to low absolute numbers of CT, NG, and having more than one concurrent vaginal infection, vaginal infections were categorized as: TV only (i.e., TV in the absence of BV, CT, NG), BV only (i.e., BV in the absence of the other infections), other infection (which included CT, NG, and any combination of multiple concurrent vaginal infection), and negative for all four infections. All patients diagnosed with an STI or symptomatic BV were prescribed treatment per standard of care. We do not have information on whether the prescribed treatment course was completed.

### Vaginal covariates

Presence of blood in the CVL was assessed on qualitative urine dip stick (positive if ≥8,000 RBCs/µL). Yeast was diagnosed by visualization of spores or pseudo hyphae on wet mount microscopy specimen treated with potassium hydroxide. The presence of semen was assessed using Abacus ABAcard p30 test for prostate-specific antigen. The examining clinician reported presence of vaginal discharge and associated color. Discharge was categorized as normal if reported as white/clear and abnormal if reported as green, yellow, brown, or other. Patient perception of abnormal discharge was evaluated by asking about presence of “thick, colored, or foul-smelling” discharge in the prior two days. Only two individuals answered in the affirmatory (one with BV one without BV). Given sparsity, this variable was not further considered in analyses.

### Statistical methods

*Sample selection.* Up to one month may be needed to clear STI DNA from the vaginal tract (Renault et al., 2011; Lazenby et al., 2017). Therefore, samples were selected from the baseline (n=58) and 3-month study visit (n=41), or from the 2nd (n=2) or 4th (n=2) visit if the baseline or 3-month samples were unavailable, respectively. *Descriptive statistics.* Each of the steps outlined below were completed separately for cytokine concentration and T cell subset (number of cells). First, we examined the natural-log transformed distribution of the inflammatory marker with boxplots with overlayed scatterplots by infection status. Kruskal-Wallis test was used to compare the distributions of cytokines and T cell subsets by infection status. Heatmaps with Spearman coefficients were generated to visualize and assess correlations between inflammatory markers. To avoid multiple comparisons among highly correlated variables, Principal Component Analysis (PCA) (Joliffe and Morgan, 1992) was used to create a cytokine inflammation score and T cell score. T cell subsets with >10% missing data were considered ineligible for analysis and excluded from the PCA (CD4+CD103+, CD4+CD45RA^hi^CCR7^lo^, CD8+CD45RA^hi^CCR7^lo^, and all six of the CD38+ cells).

The predicted value from the first principal component was then used as a continuous outcome in linear mixed regression models with a random intercept, to account for repeated measures within participants. A change in estimate approach was used for model selection (Greenland, 1989). All variables associated with the outcome at p<0.2 in bivariate analysis were entered into a full model. Variables with Wald p-value>0.05 were removed one at a time, starting with the variable explaining the least variance, and left out of the model only if removal did not change remaining estimates by ≥15%. Separate models were fit for T cells and cytokines. A sensitivity analysis was conducted excluding the second visit from n=9 individuals who were prescribed antibiotics at the baseline visit due to STI. Robust variance-covariance matrix estimation was used. All analyses were performed using Stata statistical software release 18 (StataCorp. 2023. College Station, TX).

## Results

A total of 80 individuals were enrolled in the study. Of these, 20 had no CVL samples available for analysis and were excluded, leaving an analytic sample of 60 individuals, 43 of whom had follow-up data available. Participants were median age 30 years (IQR 24, 37), half (52%) reported a household income of <$10,000 per year, and most (78%) were of self-reported Black race. Other demographic characteristics are listed in **Table 1**. At the baseline visit, there were 9 individuals (15%) with TV in the absence of co-infections, 22 (37%) with BV and no other infections, 23 (38%) who were negative for all vaginal infections (TV, CT, NG, BV), and 6 (10%) who had CT, NG, or a combination of infections. At follow-up, 5 (12%) had TV only (all were positive at baseline), 14 (33%) had BV (5 were incident among women negative for BV at baseline), 18 (42%) were negative for all four infections, and 6 (14%) had CT, NG, or a combination of infections. All participants with BV at the baseline visit were asymptomatic and none were prescribed antibiotics. Fifteen participants with TV, CT, NG, or a combination were prescribed antibiotics at the baseline visit. Of these, 6 had no follow-up visit, 8 had an infection at the follow-up visit, and 1 had no infection at follow-up. Individuals receiving antibiotics were included in the primary analysis. Given the small sample sizes, it was not possible to analyze new infections separately from ongoing/re-infections. See **Table 1**.

**Table 1:** Distribution of demographic and laboratory characteristics of patients at the baseline and follow-up visits.

Variables	Baseline Visit, N=60 n (%)	Follow-up Visit, N=43 n (%)
Age in years (median, IQR)	30 (24, 37)	n/a
Self-reported race		
Black White Multiracial	47 (78) 10 (17) 3 ( 5)	n/a
Highest educational level high school or less	31 (51)	n/a
Smoked cigarettes in the prior 6 months	32 (53)	n/a
Drank alcohol in the prior 6 months (missing, n=7)	42 (70)	n/a
Ever used crack, cocaine, heroin, or methamphetamines	9 (15)	n/a
Estimated household annual income		
<$10,000 $10,000 – $25,000 >$25,000	31 (52) 15 (25) 14 (23)	n/a
Vaginal intercourse in past month (missing, n=16)	27 (45)	26 (59)
Hormonal contraception		
None DMPA Etonogestrel implant Levonorgestrel intrauterine device	60 (100) 0 (0) 0 (0) 0 (0)	0 (0) 15 (34) 16 (36) 13 (30)
Intravaginal practice(s) in the past 1 month^1^		
Douching Use of water-based lubricant Use of oil-based lubricant Use of a feminine hygiene product	3 (5) 6 (10) 1 (2) 8 (13)	3 (7) 5 (12) 1 (2) 8 (19)
Any intravaginal practice in the prior month	15 (25)	14 (33)
*Vaginal laboratory and examination variables*		
Vaginal infection		
TV infection only BV only (Nugent score ≥7) Negative for TV, CT, NG, and BV CT, NG, or combination infection^2^	9 (15) 22 (37) 23 (38) 6 (10)	5 (12) 14 (33) 18 (42) 6 (14)
Yeast infection	3 (5)	0 (0)
Semen present in vaginal sample	11 (18)	10 (23)
Blood present in sample	4 (7)	15 (35)
Vaginal discharge on exam		
None or clear/white Brown Yellow, green, other	58 (97) 0 ( 0) 2 ( 3)	29 (67) 12 (28) 2 (5)

BV, bacterial vaginosis; CT, *Chlamydia trachomatis*; DMPA, depot-medroxyprogesterone acetate; IQR, interquartile range; NG, *Neisseria gonorrhoeae*; TV, *Trichomonas vaginalis*.

^1^Not mutually exclusive.

^2^n=9 individuals with 12 visits and the following infection/co-infections: BV/CT = 3 individuals, 4 visits; BV/NG = 2 individuals, 4 visits; TV/BV/CT = 1 individual, 1 visit; CT = 1 individual, 1 visit; BV/NG/CT = 1 individual, 1 visit; TV/CT = 1 individual, 1 visit.

n/a, not applicable.

### Comparison of cytokines and cellular immune markers by vaginal infection status

**Figure 1:**
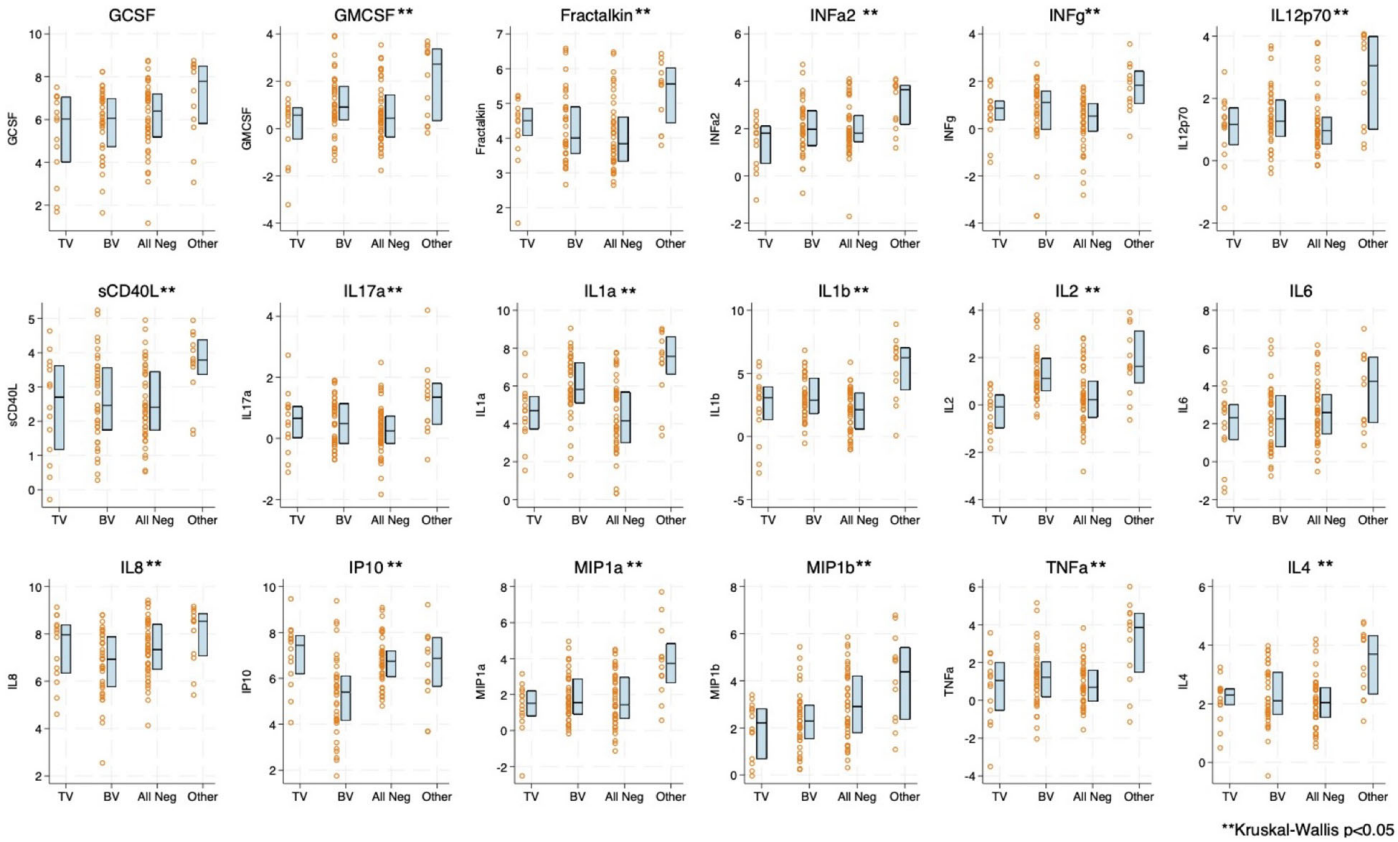
Boxplot and overlayed scatterplot of distribution of cytokine/chemokine concentration with *Trichomonas vaginalis* (TV) infection, bacterial vaginosis (BV), negative for four infections (TV, BV, *Chlamydia trachomatis* [CT], *Neisseria gonorrhea* [NG]), or Other infection (includes CT, NG, or a combination of infections). GCSF, Granulocyte colony-stimulating factor; GMCSF, Granulocyte-macrophage colony-stimulating factor; INFa2, interferon alpha-2; INFg, Interferon gamma; IP10, interferon-gamma inducible protein 10; IL, Interleukin; MIP, macrophage inflammatory protein; sCD40L, soluble CD40 ligand; and TNFa, Tumor necrosis factor alpha.

**Figure 2:**
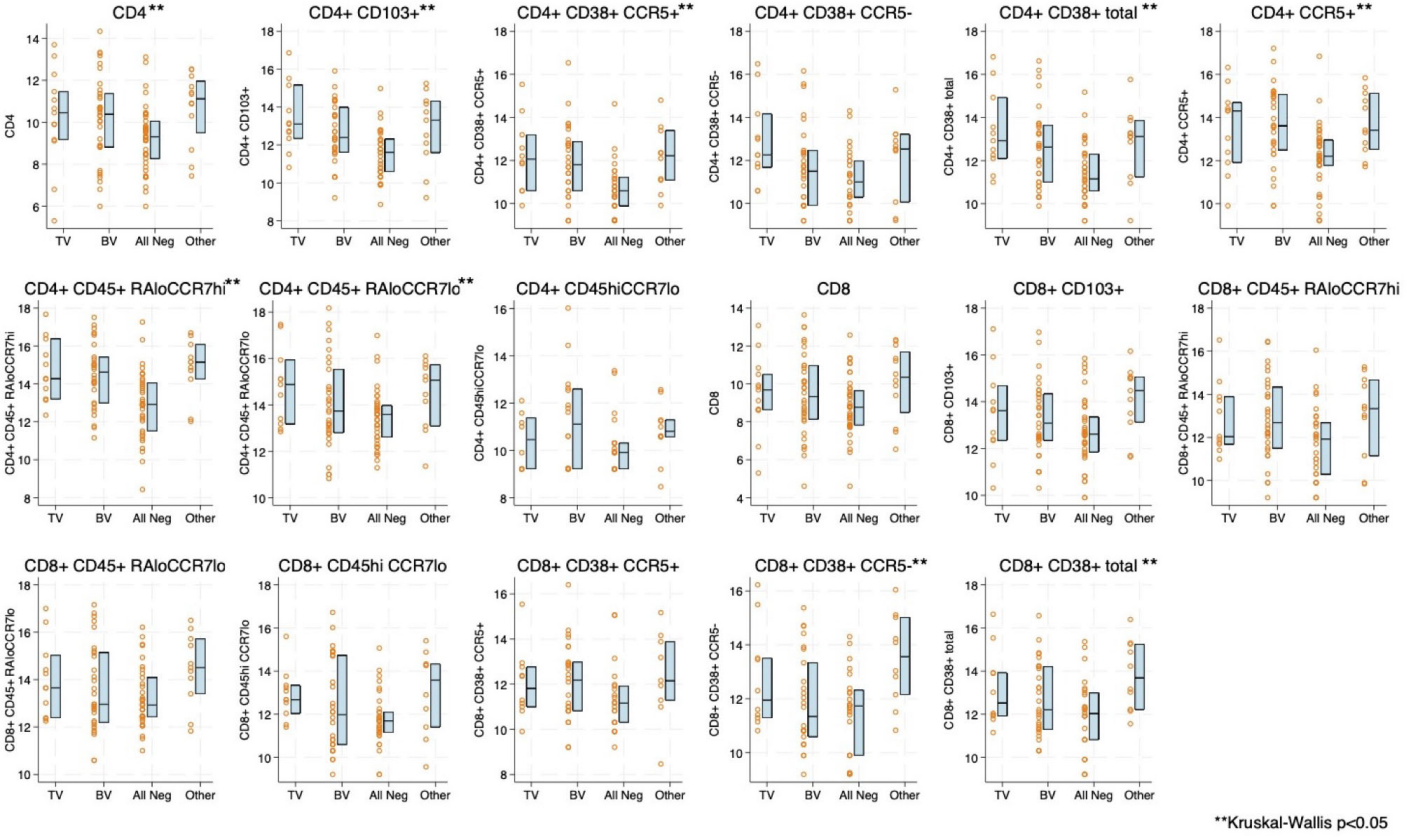
Boxplot and overlayed scatterplot of distribution of natural log-transformed T-cell subset with *Trichomonas vaginalis* (TV) infection, bacterial vaginosis (BV), negative for four infections (TV, BV, *Chlamydia trachomatis* [CT], *Neisseria gonorrhea* [NG]), or Other infection (includes CT, NG, or a combination of infections).

Boxplots of natural log transformed cytokine concentration and immune cell concentration by infection status are shown in **Figures 1**, **2**. The distribution of 16 of the 18 cytokines differed significantly by infection status. Only GCSF and IL-6 were not statistically significantly different (**Figure 1**). For the immune cells, nine of the 17 included showed statistical differences by infection, including CD4+CCR5+. Seven out of nine of the CD4+ T cell subsets differed by infection status whereas only two out of eight of the CD8+ subsets differed (**Figure 2**). Both cytokine and T cell subsets were highly positively correlated (**Supplementary Figures S1, S2**).

Variables included in the cytokine and T cell PCA models are presented in **Supplementary Table S1**. In the cytokine model, the first principal component explained 63.7% of the variance. The loadings were all positive across individual cytokines. The magnitude of the loading was <0.2 for IL1a, IL2, and IP10, indicating these influenced the PCA score less than other cytokines. In the T cell model, the first principal component explained 78.8% of the variance. The loadings in this model were all relatively high and all positive across T cell types, indicating positive correlation between the T cell type and the principal component score.

A multivariable mixed linear regression model was estimated with the predicted probability from the first principal component from the cytokine PCA model as the outcome. Adjusting for presence of blood in the sample, neither TV nor BV were associated with increased score. The “other” infection category was associated with higher score (2.60; 95%CI 0.54, 4.65). See **Table 2**.

**Table 2:** Unadjusted and adjusted estimates from: Model 1: linear mixed effects regression model with CVL cytokine score (from first principal component^1^) as outcome^2^ and Model 2: linear mixed effects regression model with Cervicovaginal lavage (CVL) T-cell score (from first principal component^3^) as outcome^4^.

Model 1: Cytokines			
Variable	UnadjustedEstimate (95%CI)	Adjusted^5^Estimate (95%CI)	P-value^6^
Vaginal Infection TV only BV only CT, NG, or combined infection Negative for NG, CT, TV, BV	-0.31 (-2.37, 1.74) 1.03 (-0.69, 2.75) 3.01 (1.12, 4.89) Ref	-0.17 (-2.20, 1.87) 0.85 (-0.57, 2.26) 2.60 (0.54, 4.65) Ref	0.06
Blood in CVL	1.78 (0.52, 3.04)	1.63 (0.48, 2.78)	0.01
Model 2: T-cells			
Variable	Unadjusted Estimate (95%CI)	Adjusted^7^ Estimate (95%CI)	P-value^6^
Vaginal Infection TV only BV only CT, NG, or combined infection Negative for NG, CT, TV, BV	1.41 (-0.57, 3.38) 1.21 (0.02, 2.41) 1.96 (0.35, 3.57) Ref	1.54 (0.00, 3.08) 0.77 (-0.24, 1.77) 1.73 (0.30, 3.16) Ref	0.02
Abnormal vaginal discharge	2.89 (2.08, 3.69)	1.55 (0.41, 2.70)	0.01
Blood in CVL	2.73 (1.74, 3.73)	2.36 (1.21, 3.52)	<0.01

^1^Principal Component model included: Ln GCSF, Ln GM-CSF, Ln Fractalkin, Ln INFa2, Ln INFg, Ln IL12p70, Ln sCD40L, Ln IL-17a, Ln IL-1a, Ln IL-1b, Ln IL-2, Ln IL-4, Ln IL-8, Ln IP-10, Ln MIP-1a, Ln MIP-1b, Ln TNFa.

^2^Covariates initially entered into full model (p<0.2 in bivariate) but later removed include: contraception, abnormal vaginal discharge. See methods section for model selection procedures.

^3^Principal Component model included: Ln number of CD4+, Ln number CD8+, Ln number CD8+ CD103+, Ln number CD4+ CCR5+, Ln number CD4+ central memory T-cells CD45RA^lo^ and CCR7^hi^, Ln number CD4+ central memory T-cells CD45RA^lo^ and CCR7^lo^, Ln number CD8+ central memory T-cells CD45RA^lo^ and CCR7^hi^, Ln number C84+ central memory T-cells CD45RA^lo^ and CCR7^lo^.

^4^Covariates with p<0.2 in bivariate analyses that were entered into full model but removed: contraception, yeast. See methods section for model selection procedures.

^5^Multivariable mixed linear regression model simultaneously adjusted for all covariates displayed plus age and income. N=60 individuals with 103 visits.

^6^Covariate p-value from adjusted model with robust variance-covariance matrix estimation.

^7^Multivariable mixed linear regression model simultaneously adjusted for all covariates displayed. N=51 individuals with 78 visits.

BV, bacterial vaginosis; CT, *Chlamydia trachomatis*; CVL, cervicovaginal lavage; NG, *Neisseria gonorrhoeae*; TV, *Trichomonas vaginalis*.

A second mixed linear regression model was estimated with T cell score (from the predicted probability from the first principal component) as the outcome. In this model, which adjusted for abnormal discharge and the presence of blood, TV infection was associated with a higher T cell score in comparison to those negative for all four infections (1.54; 95%CI 0.00, 3.08). The “other” infection category, which included CT, NG, or a combination of infections, was also associated with higher score (1.73; 95%CI 0.30, 3.16). BV was not associated with a statistically significantly higher T cell score. In a sensitivity analysis excluding the follow-up visit for the 9 individuals who received antibiotics at baseline, modeling results were substantively unchanged (**Supplementary Table S2**).

## Discussion

In this study of reproductive-aged women in Atlanta, GA we found evidence that TV infection, but not BV, was associated with the presence of mucosal vaginal T cells in comparison to those who were negative for TV, BV, NG, and CT. Those with “other” infections (CT, NG, or a combination) also had higher T cell concentration. Neither TV nor BV were associated with a score representing increased concentration of inflammatory cytokines. The vaginal environment is complex, and there has been significant heterogeneity in reported cytokine and cellular immune profiles in the presence of BV and TV (Dabee et al., 2021; Bongiorni Galego and Tasca, 2023). The immune profile can be influenced by multiple factors including individual bacterial species, viral infections, phase of the menstrual cycle, use of hormonal contraception, vaginal practices, lifestyle factors (e.g., diet, stress, smoking), host genetics, and others (Dabee et al., 2021).

We found increased T cell concentration, which included CD4+CCR5+ cells in the presence of TV infection. This has biologic plausibility, as TV infection adheres to and induces a cytotoxic response in host epithelial cells resulting in host inflammatory responses (Mercer and Johnson, 2018). Increased number of CCR5+ T cells in the presence of TV could explain the epidemiologic association between TV infection and HIV acquisition (Masha et al., 2019). In mice models, TV infection has been shown to increase trafficking of CD4+ but not CD8+ T cells into vaginal tissue (Paintlia et al., 2002; Smith and Garber, 2015). In a cross-sectional study of 65 women in Chicago (TV prevalence 32%), TV infection was *not* associated with increased expression of CD4+CCR5+ or CD8+CCR5+ T cells among women who had been exposed to HIV but remained HIV-seronegative (Jarrett et al., 2015). HIV-exposed seronegative women have previously been shown to have a relative immune quiescence (Lajoie et al., 2014) and may have differential mucosal immunologic expression in response to infectious challenge. Overall, there is a lack of published literature reporting the effect of TV infection on female genital tract T cell subsets *in vivo* (Nemati et al., 2018).

We found no difference in cytokines with BV and TV infection. Participants with CT, NG, or a combination of infections, however, did have significantly higher cytokine score and this pattern is evident across multiple individual cytokines. Sample size did not allow us to examine if a specific infection or combination of infections was driving this difference. Prior studies have shown increases in individual cytokines with TV (especially IL-8) and BV infection (multiple cytokines, often with the exception of IP-10) (Simhan et al., 2007; Jarrett et al., 2015; Dabee et al., 2021; Mtshali et al., 2021b). There is evolving understanding of BV as a heterogenous condition with differing types and concentrations of bacterial taxa present (Lev-Sagie et al., 2022), which could result in heterogeneity in cytokine signature. To avoid multiple testing with a large panel, we utilized PCA for dimension reduction. Individual cytokine differences may have been obscured by this technique. Future studies could address cytokine signatures in relation to specific vaginal infections.

The presence of blood was associated with both cellular and cytokine immune response in multivariable modeling. Presence of blood in the vaginal sample increased from 7% at baseline to 35% at follow-up despite study protocol to re-schedule clinic visits if the participant reported menses/clinically significant bleeding. This is most likely due to the initiation of progestin contraception, which increases risk of unscheduled bleeding (Zigler and McNicholas, 2017). The presence of blood in the vagina influences inflammation and infection acquisition risk. Menstrual bleeding increases risk of BV (Tamarelle et al., 2022) which, in turn, increases risk of TV acquisition (Sena et al., 2021). *Trichomonas vaginalis* infection directly damages the cervicovaginal mucosa, which could result in detection of blood in the sample (Mercer and Johnson, 2018). The presence of blood, in turn, can result in alterations in immune expression and function (Monin et al., 2020). Future research could address effects of microscopic versus macroscopic bleeding and source of bleeding (i.e., uterine, cervical, vaginal).

We utilized gram stain with Nugent scoring for BV diagnosis, which has been widely applied in research and clinical contexts (McKinnon et al., 2019). Nugent diagnosis has improved specificity in comparison to Amsel’s criteria, but symptoms of the infection do not contribute to the score (Muzny et al., 2023). All but one individual diagnosed with BV in our study would be classified as asymptomatic. Individual perception of symptoms is highly variable and many women with BV deny any symptoms. For example, in a study of nearly 3,000 women negative for CT, NG, and TV in Alabama, 58% of those with Nugent-diagnosed BV endorsed no vaginal discharge (versus 57% of women without BV) and 75% endorsed no odor (vs 82% without BV) in the prior 6 months (Klebanoff et al., 2004). Although there is controversy surrounding treatment of asymptomatic BV in a clinical context (Muzny and Schwebke, 2020), substantial evidence supports asymptomatic BV as a risk factor for the same adverse reproductive health outcomes as symptomatic BV (Myer et al., 2005; Brotman et al., 2010; Thurman and Doncel, 2011; Mlisana et al., 2012).

Strengths of this study include highly sensitive and specific diagnostic testing for STIs (nucleic acid amplification testing), longitudinal sampling, and broad assessment of immunologic markers. Importantly, the cohort under study comprises HIV negative women in Atlanta, the majority of whom identify as Black or African American; women of African descent bear disproportionate burden of HIV and STIs in the United States making this a priority population for understanding underlying drivers of increased risk for HIV/STI. Limitations include inability to assess other factors that contribute to vaginal inflammation, including the vaginal microbiome and other vaginal pathogens which have previously been shown to affect female genital tract inflammatory markers (Masson et al., 2014; Ntuli et al., 2022) (e.g., human papilloma virus, herpes simplex virus, *Mycoplasma hominis*, trichomonas vaginalis virus), although we did assess important covariates, including semen exposure and presence of blood. While the menstrual cycle phase has been shown to impact vaginal inflammatory markers (Hughes et al., 2022), the design of our study (pre/post initiation of progestin contraception) precluded control for cycle phase, although influence of contraception type was evaluated in model-building for multivariable analyses and was not statistically significant and therefore omitted from final models. Similarly, other factors known to affect the vaginal microbiome including intravaginal practices, smoking, presence of semen, and sexual behavior (Kwon and Lee, 2022) were assessed but not significant in final models.

## Conclusion

Our study found increased T cell immune activation with TV infection and “other” infection (CT, NG, or a combination of infections) versus those negative for TV, BV, CT, and NG in cis-gender women in Atlanta. These findings suggest a mechanism for increased risk of HIV acquisition with TV infection. Understanding the immunology of the vaginal mucosal environment could lead to better strategies for improving sexual and reproductive health outcomes.

## Data Availability

The raw data supporting the conclusions of this article will be made available by the authors, without undue reservation.

## References

[B1] Anderson M. R. Klink K. Cohrssen A. (2004). Evaluation of vaginal complaints. JAMA 291, 1368–1379. doi: 10.1001/jama.291.11.1368 15026404

[B2] Bongiorni Galego G. Tasca T. (2023). Infinity war: Trichomonas vaginalis and interactions with host immune response. Microb. Cell. 10, 103–116. doi: 10.15698/mic2023.05.796 37125086 PMC10140678

[B3] Brotman R. M. Klebanoff M. A. Nansel T. R. Yu K. F. Andrews W. W. Zhang J.. (2010). Bacterial vaginosis assessed by gram stain and diminished colonization resistance to incident gonococcal, chlamydial, and trichomonal genital infection. J. Infect. Dis. 202, 1907–1915. doi: 10.1086/657320 21067371 PMC3053135

[B4] Curtis K. M. Tepper N. K. Jatlaoui T. C. Berry-Bibee E. Horton L. G. Zapata L. B.. (2016). U.S. Medical eligibility criteria for contraceptive use, 2016. MMWR Recomm. Rep. 65, 1–103. doi: 10.15585/mmwr.rr6503a1 27467196

[B5] Dabee S. Barnabas S. L. Lennard K. S. Jaumdally S. Z. Gamieldien H. Balle C.. (2019). Defining characteristics of genital health in South African adolescent girls and young women at high risk for HIV infection. PloS One 14, e0213975. doi: 10.1371/journal.pone.0213975 30947260 PMC6448899

[B6] Dabee S. Passmore J. S. Heffron R. Jaspan H. B. (2021). The complex link between the female genital microbiota, genital infections, and inflammation. Infect. Immun. 89. doi: 10.1128/IAI.00487-20 PMC8091093 33558324

[B7] Fichorova R. N. Buck O. R. Yamamoto H. S. Fashemi T. Dawood H. Y. Fashemi B.. (2013). The villain team-up or how Trichomonas vaginalis and bacterial vaginosis alter innate immunity in concert. Sex Transm Infect. 89, 460–466. doi: 10.1136/sextrans-2013-051052 23903808 PMC3746192

[B8] Fichorova R. N. Trifonova R. T. Gilbert R. O. Costello C. E. Hayes G. R. Lucas J. J.. (2006). Trichomonas vaginalis lipophosphoglycan triggers a selective upregulation of cytokines by human female reproductive tract epithelial cells. Infect. Immun. 74, 5773–5779. doi: 10.1128/IAI.00631-06 16988255 PMC1594934

[B9] Flagg E. W. Meites E. Phillips C. Papp J. Torrone E. A. (2019). Prevalence of trichomonas vaginalis among civilian, noninstitutionalized male and female population aged 14 to 59 years: United States, 2013 to 2016. Sex Transm Dis. 46, e93–ee6. doi: 10.1097/OLQ.0000000000001013 31517807 PMC6924265

[B10] Gatski M. Martin D. H. Clark R. A. Harville E. Schmidt N. Kissinger P. (2011). Co-occurrence of Trichomonas vaginalis and bacterial vaginosis among HIV-positive women. Sex Transm Dis. 38, 163–166. doi: 10.1097/OLQ.0b013e3181f22f56 20842073 PMC3786582

[B11] Greenland S. (1989). Modeling and variable selection in epidemiologic analysis. Am. J. Public Health 79, 340–349. doi: 10.2105/AJPH.79.3.340 2916724 PMC1349563

[B12] Gudnadottir U. Debelius J. W. Du J. Hugerth L. W. Danielsson H. Schuppe-Koistinen I.. (2022). The vaginal microbiome and the risk of preterm birth: a systematic review and network meta-analysis. Sci. Rep. 12, 7926. doi: 10.1038/s41598-022-12007-9 35562576 PMC9106729

[B13] Haddad L. B. Swaims-Kohlmeier A. Mehta C. C. Haaland R. E. Brown N. L. Sheth A. N.. (2020). Impact of etonogestrel implant use on T-cell and cytokine profiles in the female genital tract and blood. PloS One 15, e0230473. doi: 10.1371/journal.pone.0230473 32214321 PMC7098611

[B14] Huang S. H. Hsu H. C. Lee T. F. Fan H. M. Tseng C. W. Chen I. H.. (2023). Prevalence, associated factors, and appropriateness of empirical treatment of trichomoniasis, bacterial vaginosis, and vulvovaginal candidiasis among women with vaginitis. Microbiol. Spectr. 11, e0016123. doi: 10.1128/spectrum.00161-23 37052487 PMC10269550

[B15] Hughes S. M. Levy C. N. Katz R. Lokken E. M. Anahtar M. N. Hall M. B.. (2022). Changes in concentrations of cervicovaginal immune mediators across the menstrual cycle: a systematic review and meta-analysis of individual patient data. BMC Med. 20, 353. doi: 10.1186/s12916-022-02532-9 36195867 PMC9533580

[B16] Jarrett O. D. Brady K. E. Modur S. P. Plants J. Landay A. L. Ghassemi M.. (2015). T. vaginalis Infection Is Associated with Increased IL-8 and TNFr1 Levels but with the Absence of CD38 and HLADR Activation in the Cervix of ESN. PloS One 10, e0130146. doi: 10.1371/journal.pone.0130146 26083468 PMC4470998

[B17] Joliffe I. T. Morgan B. J. (1992). Principal component analysis and exploratory factor analysis. Stat. Methods Med. Res. 1, 69–95. doi: 10.1177/096228029200100105 1341653

[B18] Kissinger P. Adamski A. (2013). Trichomoniasis and HIV interactions: a review. Sex Transm Infect. 89, 426–433. doi: 10.1136/sextrans-2012-051005 23605851 PMC3748151

[B19] Klebanoff M. A. Schwebke J. R. Zhang J. Nansel T. R. Yu K. F. Andrews W. W. (2004). Vulvovaginal symptoms in women with bacterial vaginosis. Obstet Gynecol. 104, 267–272. doi: 10.1097/01.AOG.0000134783.98382.b0 15291998

[B20] Kwon M. S. Lee H. K. (2022). Host and microbiome interplay shapes the vaginal microenvironment. Front. Immunol. 13, 919728. doi: 10.3389/fimmu.2022.919728 35837395 PMC9273862

[B21] Lajoie J. Kimani M. Plummer F. A. Nyamiobo F. Kaul R. Kimani J.. (2014). Association of sex work with reduced activation of the mucosal immune system. J. Infect. Dis. 210, 319–329. doi: 10.1093/infdis/jiu023 24421257

[B22] Lazenby G. B. Korte J. E. Tillman S. Brown F. K. Soper D. E. (2017). A recommendation for timing of repeat Chlamydia trachomatis test following infection and treatment in pregnant and nonpregnant women. Int. J. STD AIDS. 28, 902–909. doi: 10.1177/0956462416680438 27864473 PMC5798859

[B23] Lev-Sagie A. De Seta F. Verstraelen H. Ventolini G. Lonnee-Hoffmann R. Vieira-Baptista P. (2022). The vaginal microbiome: II. Vaginal dysbiotic conditions. J. Low Genit. Tract Dis. 26, 79–84. doi: 10.1097/LGT.0000000000000644 34928257 PMC8719518

[B24] Lewis F. M. T. Spicknall I. H. Flagg E. W. Papp J. R. Kreisel K. M. (2021). Incidence and prevalence of trichomonas vaginalis infection among persons aged 15 to 59 years: United States, 2018. Sex Transm Dis. 48, 232–237. doi: 10.1097/OLQ.0000000000001383 33492095 PMC10240849

[B25] Masha S. C. Cools P. Sanders E. J. Vaneechoutte M. Crucitti T. (2019). Trichomonas vaginalis and HIV infection acquisition: a systematic review and meta-analysis. Sex Transm Infect. 95, 36–42. doi: 10.1136/sextrans-2018-053713 30341233 PMC6580735

[B26] Masson L. Mlisana K. Little F. Werner L. Mkhize N. N. Ronacher K.. (2014). Defining genital tract cytokine signatures of sexually transmitted infections and bacterial vaginosis in women at high risk of HIV infection: a cross-sectional study. Sex Transm Infect. 90, 580–587. doi: 10.1136/sextrans-2014-051601 25107710

[B27] McKinnon L. R. Achilles S. L. Bradshaw C. S. Burgener A. Crucitti T. Fredricks D. N.. (2019). The evolving facets of bacterial vaginosis: implications for HIV transmission. AIDS Res. Hum. Retroviruses 35, 219–228. doi: 10.1089/aid.2018.0304 30638028 PMC6434601

[B28] Mercer F. Johnson P. J. (2018). Trichomonas vaginalis: pathogenesis, symbiont interactions, and host cell immune responses. Trends Parasitol. 34, 683–693. doi: 10.1016/j.pt.2018.05.006 30056833 PMC11132421

[B29] Mitchell C. Marrazzo J. (2014). Bacterial vaginosis and the cervicovaginal immune response. Am. J. Reprod. Immunol. 71, 555–563. doi: 10.1111/aji.2014.71.issue-6 24832618 PMC4128638

[B30] Mlisana K. Naicker N. Werner L. Roberts L. van Loggerenberg F. Baxter C.. (2012). Symptomatic vaginal discharge is a poor predictor of sexually transmitted infections and genital tract inflammation in high-risk women in South Africa. J. Infect. Dis. 206, 6–14. doi: 10.1093/infdis/jis298 22517910 PMC3490689

[B31] Monin L. Whettlock E. M. Male V. (2020). Immune responses in the human female reproductive tract. Immunology 160, 106–115. doi: 10.1111/imm.v160.2 31630394 PMC7218661

[B32] Mtshali A. Ngcapu S. Mindel A. Garrett N. Liebenberg L. (2021a). HIV susceptibility in women: The roles of genital inflammation, sexually transmitted infections and the genital microbiome. J. Reprod. Immunol. 145, 103291. doi: 10.1016/j.jri.2021.103291 33647576

[B33] Mtshali A. San J. E. Osman F. Garrett N. Balle C. Giandhari J.. (2021b). Temporal changes in vaginal microbiota and genital tract cytokines among South African women treated for bacterial vaginosis. Front. Immunol. 12, 730986. doi: 10.3389/fimmu.2021.730986 34594336 PMC8477043

[B34] Muzny C. A. Cerca N. Elnaggar J. H. Taylor C. M. Sobel J. D. van der Pol B. (2023). State of the art for diagnosis of bacterial vaginosis. J. Clin. Microbiol. 61, e0083722. doi: 10.1128/jcm.00837-22 37199636 PMC10446871

[B35] Muzny C. A. Schwebke J. R. (2020). Asymptomatic bacterial vaginosis: to treat or not to treat? Curr. Infect. Dis. Rep. 22. doi: 10.1007/s11908-020-00740-z PMC8015387 33814990

[B36] Myer L. Denny L. Telerant R. Souza M. Wright T. C. Jr. Kuhn L. (2005). Bacterial vaginosis and susceptibility to HIV infection in South African women: a nested case-control study. J. Infect. Dis. 192, 1372–1380. doi: 10.1086/jid.2005.192.issue-8 16170754

[B37] Nemati M. Malla N. Yadav M. Khorramdelazad H. Jafarzadeh A. (2018). Humoral and T cell-mediated immune response against trichomoniasis. Parasite Immunol. 40. doi: 10.1111/pim.2018.40.issue-3 29266263

[B38] Ntuli L. Mtshali A. Mzobe G. Liebenberg L. J. Ngcapu S. (2022). Role of immunity and vaginal microbiome in clearance and persistence of human papillomavirus infection. Front. Cell Infect. Microbiol. 12, 927131. doi: 10.3389/fcimb.2022.927131 35873158 PMC9301195

[B39] Paintlia M. K. Kaur S. Gupta I. Ganguly N. K. Mahajan R. C. Malla N. (2002). Specific IgA response, T-cell subtype and cytokine profile in experimental intravaginal trichomoniasis. Parasitol. Res. 88, 338–343. doi: 10.1007/s004360100396 11999021

[B40] Peebles K. Velloza J. Balkus J. E. McClelland R. S. Barnabas R. V. (2019). High global burden and costs of bacterial vaginosis: A systematic review and meta-analysis. Sex Transm Dis. 46, 304–311. doi: 10.1097/OLQ.0000000000000972 30624309

[B41] Ravel J. Moreno I. Simon C. (2021). Bacterial vaginosis and its association with infertility, endometritis, and pelvic inflammatory disease. Am. J. Obstet Gynecol. 224, 251–257. doi: 10.1016/j.ajog.2020.10.019 33091407

[B42] Renault C. A. Israelski D. M. Levy V. Fujikawa B. K. Kellogg T. A. Klausner J. D. (2011). Time to clearance of Chlamydia trachomatis ribosomal RNA in women treated for chlamydial infection. Sex Health 8, 69–73. doi: 10.1071/SH10030 21371385

[B43] Sena A. C. Goldstein L. A. Ramirez G. Parish A. J. McClelland R. S. (2021). Bacterial vaginosis and its association with incident trichomonas vaginalis infections: A systematic review and meta-analysis. Sex Transm Dis. 48, e192–e201. doi: 10.1097/OLQ.0000000000001537 34433796 PMC8594503

[B44] Shannon B. Gajer P. Yi T. J. Ma B. Humphrys M. S. Thomas-Pavanel J.. (2017). Distinct effects of the cervicovaginal microbiota and herpes simplex type 2 infection on female genital tract immunology. J. Infect. Dis. 215, 1366–1375. doi: 10.1093/infdis/jix088 28201724 PMC5451606

[B45] Simhan H. N. Anderson B. L. Krohn M. A. Heine R. P. Martinez de Tejada B. Landers D. V.. (2007). Host immune consequences of asymptomatic Trichomonas vaginalis infection in pregnancy. Am. J. Obstet Gynecol. 196, 59 e1–59 e5. doi: 10.1016/j.ajog.2006.08.035 17240235

[B46] Smith J. D. Garber G. E. (2015). Trichomonas vaginalis infection induces vaginal CD4+ T-cell infiltration in a mouse model: a vaccine strategy to reduce vaginal infection and HIV transmission. J. Infect. Dis. 212, 285–293. doi: 10.1093/infdis/jiv036 25616405

[B47] Swaims-Kohlmeier A. Haaland R. E. Haddad L. B. Sheth A. N. Evans-Strickfaden T. Lupo L. D.. (2016). Progesterone levels associate with a novel population of CCR5+CD38+ CD4 T cells resident in the genital mucosa with lymphoid trafficking potential. J. Immunol. 197, 368–376. doi: 10.4049/jimmunol.1502628 27233960 PMC4912879

[B48] Tamarelle J. Shardell M. D. Ravel J. Brotman R. M. (2022). Factors associated with incidence and spontaneous clearance of molecular-bacterial vaginosis: results from a longitudinal frequent-sampling observational study. Sex Transm Dis. 49, 649–656. doi: 10.1097/OLQ.0000000000001662 35969846 PMC9387550

[B49] Thurman A. R. Doncel G. F. (2011). Innate immunity and inflammatory response to Trichomonas vaginalis and bacterial vaginosis: relationship to HIV acquisition. Am. J. Reprod. Immunol. 65, 89–98. doi: 10.1111/j.1600-0897.2010.00902.x 20678168

[B50] Thurman A. R. Kimble T. Herold B. Mesquita P. M. Fichorova R. N. Dawood H. Y.. (2015). Bacterial vaginosis and subclinical markers of genital tract inflammation and mucosal immunity. AIDS Res. Hum. Retroviruses 31, 1139–1152. doi: 10.1089/aid.2015.0006 26204200 PMC4651020

[B51] Van Gerwen O. T. Craig-Kuhn M. C. Jones A. T. Schroeder J. A. Deaver J. Buekens P.. (2021). Trichomoniasis and adverse birth outcomes: a systematic review and meta-analysis. BJOG 128, 1907–1915. doi: 10.1111/1471-0528.16774 34036690 PMC9704755

[B52] Wiringa A. E. Ness R. B. Darville T. Beigi R. H. Haggerty C. L. (2019). Trichomonas vaginalis, endometritis and sequelae among women with clinically suspected pelvic inflammatory disease. Sex Transm Infect 96, 436–438. doi: 10.1136/sextrans-2019-054079 31719170

[B53] Zigler R. E. McNicholas C. (2017). Unscheduled vaginal bleeding with progestin-only contraceptive use. Am. J. Obstet Gynecol. 216, 443–450. doi: 10.1016/j.ajog.2016.12.008 27988268

