# Supplementary material for: Cytokine concentration and T cell subsets in the female genital tract in the presence of bacterial vaginosis and Trichomonas vaginalis
Source: Front Cell Infect Microbiol. 2025 Apr 17;15:1539086. doi: 10.3389/fcimb.2025.1539086 (PMC12043704; doi:10.3389/fcimb.2025.1539086)
Supplement: Supplementary file 1 [file DataSheet1.pdf]

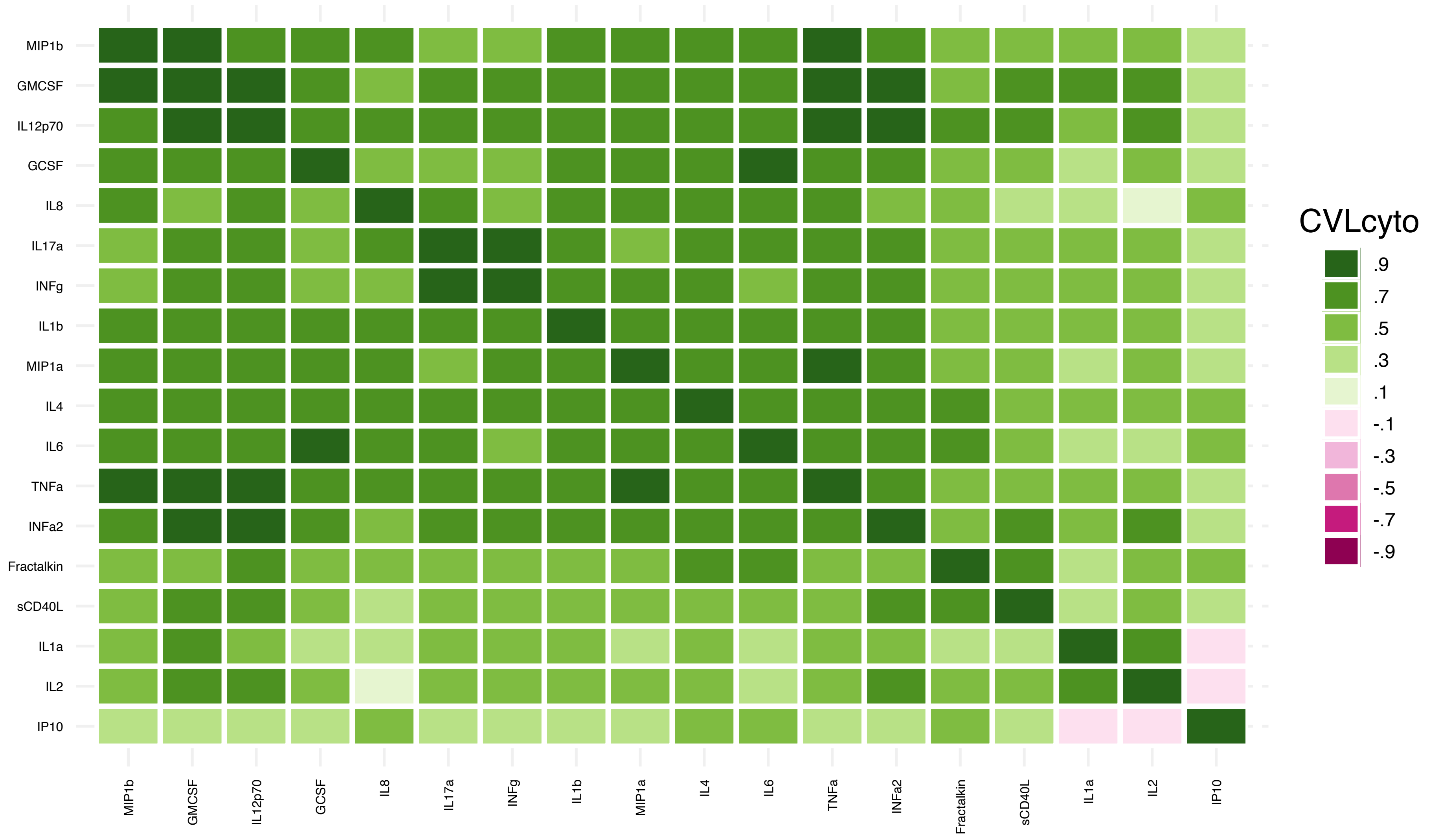

Supplemental Figure 1. Heatmap showing Spearman correlation coefficients between cytokines in cervicovaginal lavage supernatant. Abbreviations: GCSF, Granulocyte colony-stimulating factor; GMCSF, Granulocyte-macrophage colony-stimulating factor; INFa2, interferon alpha-2; INFg, Interferon gamma; IP10, interferon-gamma inducible protein 10; IL, Interleukin; MIP, macrophage inflammatory protein; sCD40L, soluble CD40 ligand; and TNFa, Tumor necrosis factor alpha
