# Supplementary material for: Cytokine concentration and T cell subsets in the female genital tract in the presence of bacterial vaginosis and Trichomonas vaginalis
Source: Front Cell Infect Microbiol. 2025 Apr 17;15:1539086. doi: 10.3389/fcimb.2025.1539086 (PMC12043704; doi:10.3389/fcimb.2025.1539086)
Supplement: Supplementary file 2 [file DataSheet2.pdf]

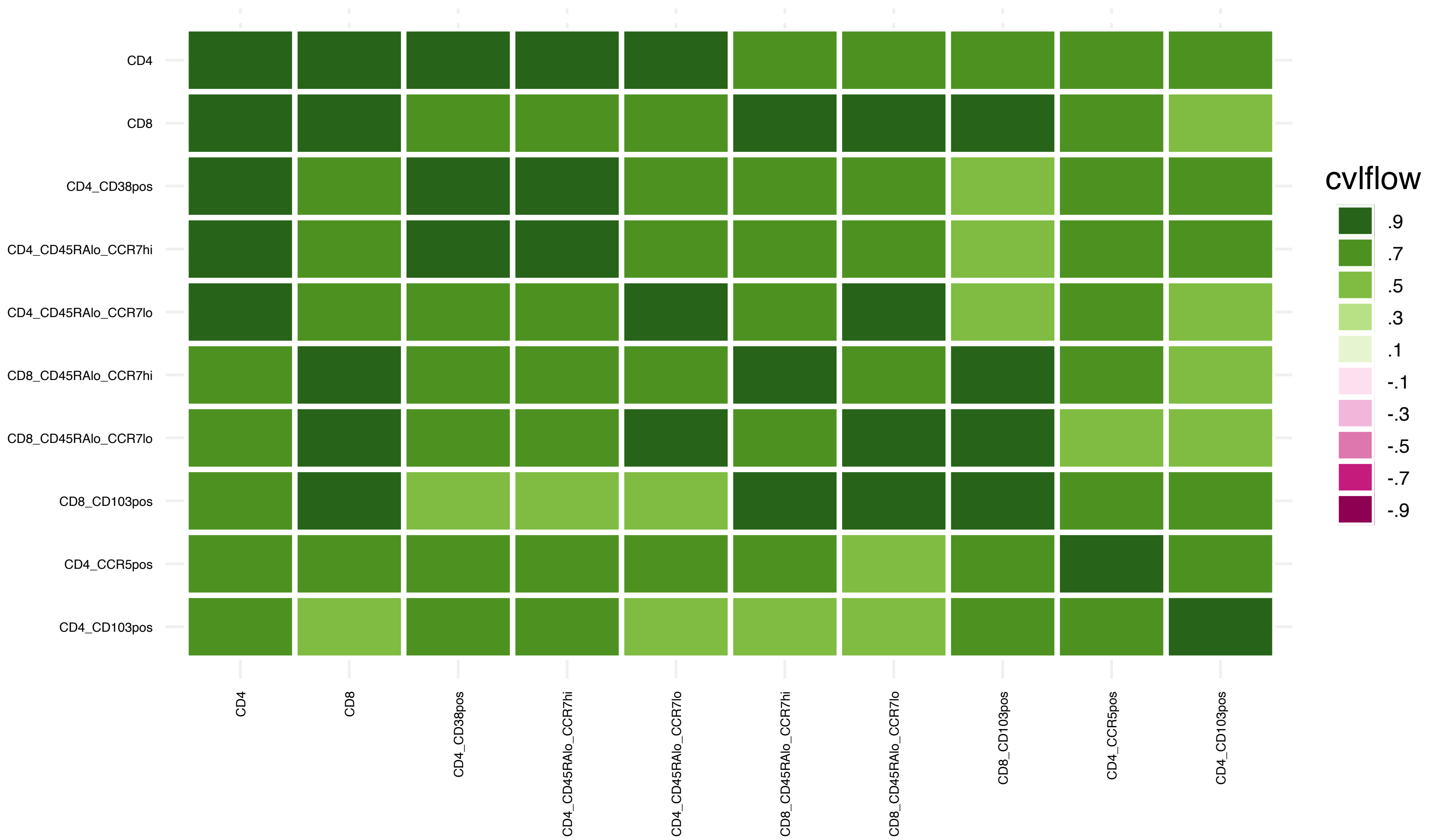

Supplemental Figure 2. Heatmap showing Spearman correlation coefficients between T-cell subsets in the female genital tract
