# Supplementary material for: Cytokine concentration and T cell subsets in the female genital tract in the presence of bacterial vaginosis and Trichomonas vaginalis
Source: Front Cell Infect Microbiol. 2025 Apr 17;15:1539086. doi: 10.3389/fcimb.2025.1539086 (PMC12043704; doi:10.3389/fcimb.2025.1539086)
Supplement: Supplementary file 3 [file Table1.docx]

Supplemental Table 1: Proportion explained, eigenvalue and loadings of first principal component from two principal component models

| **Flow Cytometry Model**  Proportion Explained by 1^st^ principal component: 78.8%  1^st^ principal component Eigenvalue: 6.30 | |
| --- | --- |
| **Variable^1^** | **Loading on 1^st^ Principal Component** |
| CD4+ | 0.3703 |
| CD8+ | 0.3816 |
| CD8+ CD103+ | 0.3318 |
| CD4+ CCR5+ | 0.3261 |
| CD4+ central memory T-cells CD45RA^lo^ and CCR7^hi^ | 0.3507 |
| CD4+ central memory T-cells CD45RA^lo^ and CCR7^lo^ | 0.3479 |
| CD8+ central memory T-cells CD45RA^lo^ and CCR7^hi^ | 0.3541 |
| C84+ central memory T-cells CD45RA^lo^ and CCR7^lo^ | 0.3625 |
| **Cytokine Model**  Proportion Explained by 1^st^ principal component: 63.7%  1^st^ principal component Eigenvalue: 11.5 | |
| **Variable^1^** | **Loading on 1^st^ Principal Component** |
| GCSF | 0.2416 |
| GMCSF | 0.2720 |
| Fractalkin | 0.2178 |
| INFa2 | 0.2508 |
| INFg | 0.2327 |
| IL12p70 | 0.2736 |
| sCD40L | 0.2154 |
| IL17a | 0.2300 |
| IL1a | 0.1764 |
| IL1b | 0.2471 |
| IL2 | 0.1966 |
| IL4 | 0.2677 |
| IL6 | 0.2530 |
| IL8 | 0.2211 |
| IP10 | 0.1357 |
| MIP1a | 0.2459 |
| MIP1b | 0.2588 |
| TNFa | 0.2603 |

^1^Ln of number of cells or of cytokine/chemokine used in PCA model
