# Supplementary material for: Cytokine concentration and T cell subsets in the female genital tract in the presence of bacterial vaginosis and Trichomonas vaginalis
Source: Front Cell Infect Microbiol. 2025 Apr 17;15:1539086. doi: 10.3389/fcimb.2025.1539086 (PMC12043704; doi:10.3389/fcimb.2025.1539086)
Supplement: Supplementary file 4 [file Table2.docx]

Supplemental Table 2: Unadjusted and adjusted estimates from linear mixed effects regression models with Cervicovaginal lavage (CVL) T-cell score (from first principal component^1^) as outcome, excluding the second visit from n=9 individuals who received antibiotics at baseline

| Variable | Unadjusted  Estimate (95%CI) | Adjusted^2^  Estimate (95%CI) | p-value^3^ |
| --- | --- | --- | --- |
| Vaginal Infection  TV only  BV only  CT, NG, or combined infection  Negative for NG, CT, TV, BV | 1.36 (-0.59, 3.31)  1.21 (0.00, 2.42)  1.68 (-0.30, 3.66)  Ref | 2.03 (0.11, 3.96)  0.78 (-0.23, 1.80)  1.32 (-0.36, 2.99)  Ref | 0.06 |
| Abnormal vaginal discharge | 2.95 (1.99, 3.91) | 1.41 (0.09, 2.73) | 0.04 |
| Blood in CVL | 2.73 (1.61, 3.84) | 2.50 (1.22, 3.77) | <0.01 |

BV, bacterial vaginosis; CT, *Chlamydia trachomatis*; CVL, cervicovaginal lavage; NG, *Neisseria gonorrhoeae*; TV, *Trichomonas vaginalis*

^1^Principal Component model included: Ln number of CD4+, Ln number CD8+, Ln number CD8+ CD103+, Ln number CD4+ CCR5+, Ln number CD4+ central memory T-cells CD45RA^lo^ and CCR7^hi^, Ln number CD4+ central memory T-cells CD45RA^lo^ and CCR7^lo^, Ln number CD8+ central memory T-cells CD45RA^lo^ and CCR7^hi^, Ln number C84+ central memory T-cells CD45RA^lo^ and CCR7^lo^

^2^Multivariable mixed linear regression model simultaneously adjusted for all covariates displayed. N=50 individuals with 70 visits.

^3^Covariate p-value from adjusted model with robust variance-covariance matrix estimation.
